# Carcinogenesis in the Pituitary Dwarf Mouse. The Response to 2-Aminofluorene

**DOI:** 10.1038/bjc.1960.23

**Published:** 1960-06

**Authors:** F. Bielschowsky, Marianne Bielschowsky

## Abstract

**Images:**


### CARCINOGENESIS IN THE PITUITARY DWARF MOUSE. THE RESPONSE TO 2-AMINOFLUORENE

F. BIELSCHOWSKY AND MARIANNE BIELSCHOWSKY

*From the Hugh Adam Cancer Research Department of the Medical School and the New Zealand Branch of the British Empire Cancer Campaign, University of Otago, Dunedin, New Zealand*

Received for publication April 23, 1960

THE connective tissue elements of pituitary dwarf mice have been found to respond to a subcutaneous injection of 0·5 mg. of methylcholanthrene in the same way as those of their normal sized litter mates.

The purpose of the experiment described in this paper was to compare the susceptibility of these mice to a systemically acting carcinogenic agent, 2-aminofluorene (AF), which affects not only the parenchymal cells of a variety of organs such as liver and mammary glands, but induces also neoplastic changes in the transitional epithelium of the urinary tract.

Bickis, Estwick and Campbell (1956) did not find any spontaneous tumours in their colony of pituitary dwarf mice. Nevertheless it was felt necessary to obtain some information on the incidence of spontaneous neoplasms in old dwarfs and in the phenotypically normal heterozygotes of our stock, so that induced tumours might be distinguished from spontaneous ones. Therefore untreated animals carrying the dwarf gene were used as additional controls.

Some preliminary results of our investigation have been mentioned at the Symposium on Functional Components of Carcinogenesis held at Rehovoth in 1959.

### MATERIALS AND METHODS

The origin of the mice and their maintenance have been described in a previous paper (Bielschowsky and Bielschowsky, 1959).

Whenever possible the experimental dwarfs were matched with normal sized litter mates of the same sex.

Painting of the interscapular region with a 4 per cent solution of AF in acetone was started when the mice were 8–12 weeks old. Ninety applications were given; a No. 4 brush was used for the dwarfs and a No. 6 for the normal sized animals. The total dose administered to the former was approximately 135 mg. and to the latter approximately 270 mg.

The animals were killed when a palpable tumour was present or when decline in health made it advisable, and the remainder at the 52nd week of the experiment. Their maximum age was 14½ months.

The untreated animals were kept in a separate room under the same conditions as the experimental mice. The dwarfs were killed when they were 21–25 months old. The normal sized mice were known to be heterozygotes, the majority being parents of the dwarfs treated with AF. Some of these heterozygotes had to be killed during the 13th–18th month of life because they were seriously ill, but most of them were more than 19 months old when autopsied.

The post mortem examinations of the dwarfs were carried out under a magnifying glass. The histological methods used were those previously described (Bielschowsky and Bielschowsky, 1959).

### RESULTS

The average weight of the dwarfs at the start of the experiment was 7 g. and 8·5 g. at the time when treatment with AF was stopped. The corresponding values for the normal sized mice were 23 and 26 g. Since the amount of AF given to the dwarfs was approximately 50 per cent of that given to their phenotypically normal litter mates the former received, per gram body weight, more than the latter. Of the 55 dwarfs treated with the carcinogen, 39 survived for at least 29 weeks, the time when the first tumour, a cancer of the bladder, was found. All other tumours observed in this group occurred in animals killed in the 39th–52nd week of the experiment.

In the normal sized litter mates the earliest tumour, also a cancer of the bladder, was found in the 34th week. The average age of these mice at death was 4½ weeks less than that of the dwarfs.

Table I shows the neoplastic lesions found at autopsy in untreated controls (Groups I and II) and Table II those seen in the mice treated with AF (Groups III and IV). A comparison of the tumour incidence in the four groups reveals differences between the dwarfs (Groups I and III) and the phenotypically normal mice (Groups II and IV) in respect to both spontaneous and induced tumours. For instance, in the 41 normal sized mice treated with AF (Group III) 32 hepatomas were found, but only 13 liver tumours in the 39 dwarfs of group IV. There was therefore a highly significant association of normal body size with hepatoma formation ($P < 0{\cdot}001$). In the dwarfs both sexes were equally affected. In group III however there was a prevalence of hepatomas in the females, 90 per cent of

TABLE I.—*Neoplastic Lesions Found at Autopsy in Untreated Controls*

| | Control animals | |
|---|---|---|
| | Group I | Group II |
| | Neoplastic lesions found in | |
| | Normal sized untreated | Dwarfs untreated |
| Total number of animals | 28 (♂ 14, ♀ 14) | 37 (♂ 17, ♀ 20) |
| Age in months | 13–25 | 21½–25½ |
| Animals with tumours in— | | |
| Liver | — | 3 |
| Lung | 11 | — |
| Duodenum | 4 | 1 |
| Breast | 2 | — |
| Pituitary | 2 | — |
| Ovary | 1 | 2 |
| Lymph glands | 1 | — |

which developed tumours of the liver, whereas the incidence in the males of this group was 65 per cent.

The first palpable hepatomas occurred in 2 normal sized females during the 37th week of the expriment. Two weeks later a male dwarf of group IV was found to have a liver tumour with a diameter of 3 mm. Three blocks were taken from

TABLE II.—*Neoplastic Lesions Seen in Mice Treated with AF*

| | Experimental animals | |
|---|---|---|
| | Group III | Group IV |
| | Neoplastic lesions found in | |
| | Normal sized treated with A.F. | Dwarfs treated with A.F. |
| Total number of animals | 41 (♂ 20, ♀ 21) | 39 (♂ 17, ♀ 22) |
| Duration of experiment (weeks) | 34–52 | 29–52 |
| Animals with tumours in— | | |
| Liver | 32 (♂ 13, ♀ 19) | 13 (♂ 7, ♀ 6) |
| Lung | 5 | — |
| Pyloric region | 12 | 3 |
| Breast | 8 | — |
| Bladder | 4 | 4 |
| Kidney | 1 | — |
| Ovary | 1 | — |
| Uterus | 1 | — |
| Gall bladder | 2 | — |

those livers of mice of groups III and IV which at post mortem appeared normal. Histological investigation failed to reveal neoplastic changes in those of normal sized mice but in 2 of the dwarfs minute lesions were seen. They were formed by the same type of cell depicted in Fig. 1, a photomicrograph of a macroscopically recognizable hepatoma. This indicates that more hepatomas might have developed in the dwarfs treated with AF had these animals been observed for a longer period. Histologically there were no essential differences between the liver tumours found in groups III and IV, but in the latter, occasionablly foci of regressing neoplastic lesions (Fig. 3) were seen in livers which at the same time contained progressing tumours (Fig. 1 and 2). Only once, in a dwarf, were deposits of hepatoma cells detected in the lungs (Fig. 4).

Spontaneous liver tumours were not found in untreated heterozygotes, but 3 of the dwarfs of group II had one or more macroscopically recognizable nodules in the liver. Only in one instance did a neoplastic lesion resemble those seen in the liver of the experimental animals (Fig. 5). In the 2 others the histological picture was of a more benign nature. One old dwarf had a single yellowish coloured nodule composed of pale plant-like cells (Fig. 6), the other had 5 distinct nodules the cells of which differed only slightly, by the basophilia of the cytoplasm, from the surrounding liver cells. In this connection it seems worth mentioning that areas of necrosis were not infrequent in the livers of old untreated dwarfs, but amyloid present in some livers of untreated heterozygotes was never seen.

Benign papillomas of the gall bladder (Fig. 7) occurred in 2 of the normal sized mice treated with AF.

No breast tumours developed in the dwarfs of groups II or IV, but 2 spontaneous mammary cancers were seen in the untreated heterozygotes and 8 occurred in the normal sized mice treated with AF. The spontaneous tumours were typical alveolar carcinomas and all the latter contained acanthotic areas.

There was little difference between the dwarfs and their litter mates in the response of the transitional epithelium to AF. In 4 animals of each group macroscopically recognizable bladder tumours were seen. Three were classified as malignant because the muscular layer of the bladder was deeply invaded. One such cancer occurred in a dwarf. In addition, there was one carcinoma of the pelvis in an animal of group III. No spontaneous tumours of the urinary tract were found in the untreated mice, but benign papillomas of the duodenum occurred in a few mice of groups I and II. The incidence of these lesions was considerably higher in the animals treated with AF. In the heterozygotes of group III 12 tumours of the intestinal tract, situated mainly on either side of the pylorus, were found. Two of these tumours had invaded the muscular layer (Fig. 8). On the whole they were considerably larger than the spontaneous ones and the same holds true for the three benign papillomas found in the dwarfs treated with AF (Fig. 9 and 10).

Tumours of the lung were not found in any of the dwarfs of either group II or IV although this was the most common spontaneous tumour in the heterozygotes, 40 per cent of which had single pulmonary adenomas. Of the normal sized mice treated with AF, 5 developed benign lung tumours. In 3 of these animals they were multiple but were of a smaller size than those observed in the much older mice of group I.

All the ovarian tumours found, including the one seen in an animal of group III, are considered to be spontaneous benign neoplasms. Three of them were granulosa cell tumours and the fourth appeared to arise from the cells of the ovarian stroma.

### DISCUSSION

In the rat ablation of thyroid (Bielschowsky and Hall, 1953) or pituitary (O'Neal, Hoffman, Dodge and Griffin, 1958) prevents the development of hepatomas induced by AF or related compounds, but the liver of the pituitary dwarf mouse is susceptible to the action of this aromatic amine. In spite of the absence of growth hormone and in spite of an extremely low level of thyroid function, the liver cells of the dwarf react to the carcinogen qualitatively in a similar manner as those of normal mice or intact rats. The development of hepatomas is slowed down, but not inhibited. In conjunction with these findings the occurrence of spontaneous liver tumours in 2-year-old dwarfs is of considerable interest and stresses the fact that in pituitary dwarf mice, at least, a multiple hormone deficiency does not prevent neoplastic growth in the liver.

The failure of the female dwarfs to develop breast tumours is easily explained by the virtual absence of mammary gland tissue ; they do not even have nipples. Although gonadotrophs are present in the pituitary of the dwarf mouse there is hardly any indication of oestrogen secretion by the ovaries. Uterus and vagina always showed the picture of sexual immaturity ; also in our material there was

---

### EXPLANATION OF PLATES

FIG. 1.—Hepatoma formed by large cells with eosinophilic cytoplasm from a dwarf of group IV. H. and E. ×80.
FIG. 2.—Another hepatoma from the same dwarf as Fig. 1. H. and E. ×80.
FIG. 3.—A regressing neoplastic lesion—same dwarf as Fig. 1 and 2. H. and E. ×80.
FIG. 4.—Hepatoma cells in lung of a dwarf of group IV. H. and E. ×80.
FIG. 5.—Spontaneous hepatoma from a dwarf of group II. H. and E. ×80.
FIG. 6.—Benign nodule from dwarf of group II. H. and E. ×80.
FIG. 7.—Benign tumour of the gall bladder from a normal sized mouse of group III. H. and E. ×80.
FIG. 8.—Adenocarcinoma of small intestine from a normal sized mouse of group III. H. and E. ×20.
FIG. 9.—Papilloma of small intestine from a dwarf of group IV. H. and E. ×20.
FIG. 10.—Papilloma of duodenum from another dwarf of group IV. H. and E. ×20.

1

2

3

4

5

Bielschowsky and Bielschowsky.

6

7

8

9

10

never any sign of luteinization in the ovaries. Therefore the four hormones which normally stimulate the mammary glands, oestrogen, progesterone, prolactin and growth hormone, if present at all, were secreted in only extremely low quantities. In these animals the only evidence for gonadotrophic action was the size of the follicles. Some were larger than those seen in the gonads of females hypophysectomized when sexually immature, but they never reached the size and appearance of mature follicles. Nevertheless in 2 ovaries of old untreated dwarfs non-functional neoplastic lesions were found. In this context it might be mentioned that in the male dwarfs evidence for gonadotrophic stimulation was more obvious. The testes of some dwarfs had descended into the scrotum and in such glands sperm was produced. However, stimulated secondary sex organs were rarely seen in a male dwarf.

In view of the occurrence of spontaneous benign papillomas of the small intestine in both groups of untreated animals it seems doubtful that the similar tumours observed in mice of groups III and IV were induced by AF. The higher incidence of these neoplasms, the larger size and the invasiveness of some of them suggests that the carcinogen accelerated their development. In the dwarfs this process seems to progress more slowly than in the normal sized animals ($P = 0{\cdot}013$).

The absence of lung tumours in the dwarfs is surprising because in the normal sized animals these neoplasms were far from infrequent. Of 17 untreated heterozygotes, older than 20 months, 9 had pulmonary adenomas. It is well established that the genetic constitution determines the susceptibility of mice to the development of spontaneous as well as induced adenomas of the lung (Heston, 1942), whereas, as far as we are aware, there is no evidence for hormones playing an important role in their pathogenesis. Still, in view of the close relationship of the experimental mice of groups III and IV which were litter mates, and the untreated heterozygotes of group I which were their parents, it seems difficult to believe that non-systemic genetic factors could account for the difference in incidence.

Further experiments are under way to test whether or not it is possible to obtain lung tumours in the pituitary dwarf mice of our stock with the aid of other carcinogenic agents.

### SUMMARY

The susceptibility of pituitary dwarf mice to 2-aminofluorene was compared with that of their normal sized litter mates.

In the dwarfs the transitional epithelium of the bladder was found to be as sensitive as that of the phenotypically normal animals, but, in the former, liver cells and the epithelium of the small intestine reacted in a significantly slower manner to the carcinogen.

No tumours of the breast or lungs were found in the dwarfs whether treated or untreated. In the closely related normal sized animals these organs exhibited benign and malignant neoplasms.

### REFERENCES

BICKIS, I., ESTWICK, R. R. AND CAMPBELL, J. S.—(1956) *Cancer*, **9**, 763.
BIELSCHOWSKY, F. AND BIELSCHOWSKY, M.—(1959) *Brit. J. Cancer*, **13**, 302.
*Idem* AND HALL, W. H.—(1953) *Ibid.*, **7**, 358.
HESTON, W. E.—(1942) *J. nat. Cancer Inst.*, **3**, 79.
O'NEAL, M. A., HOFFMAN, H. E., DODGE, B. G. AND GRIFFIN, A. C.—(1958) *Ibid.*, **21**, 1161.


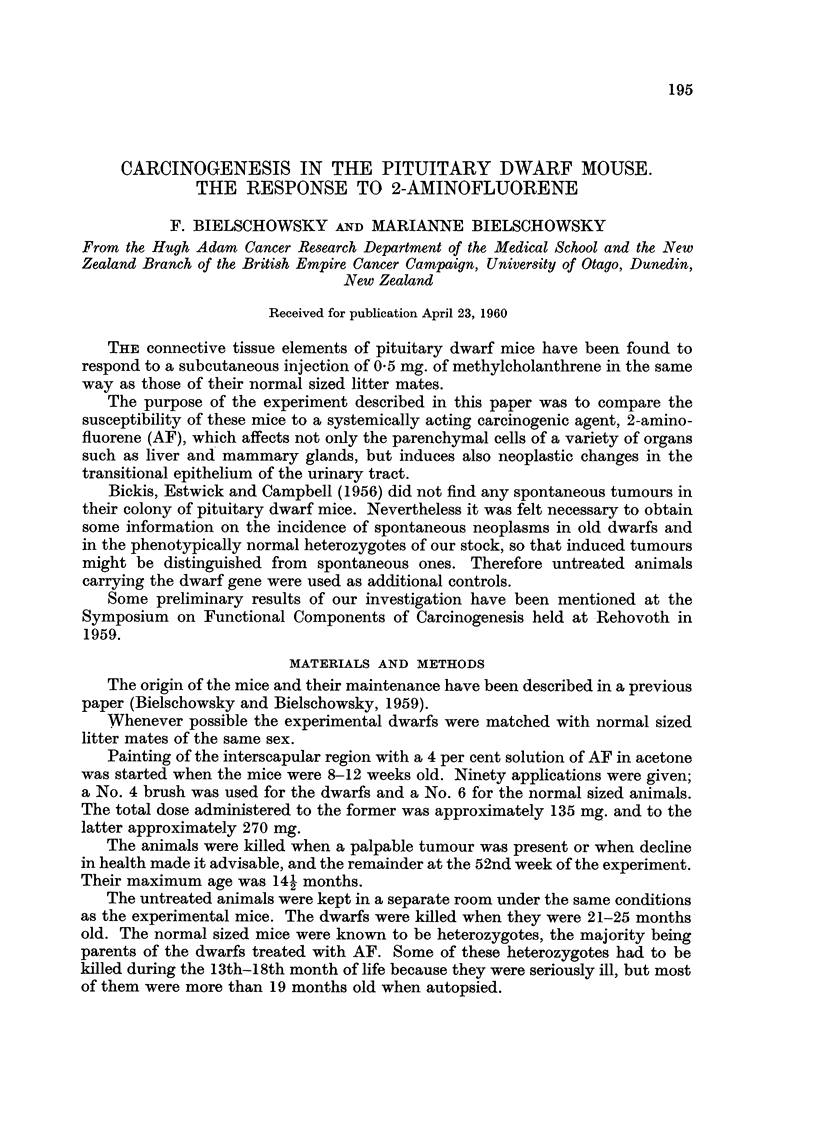

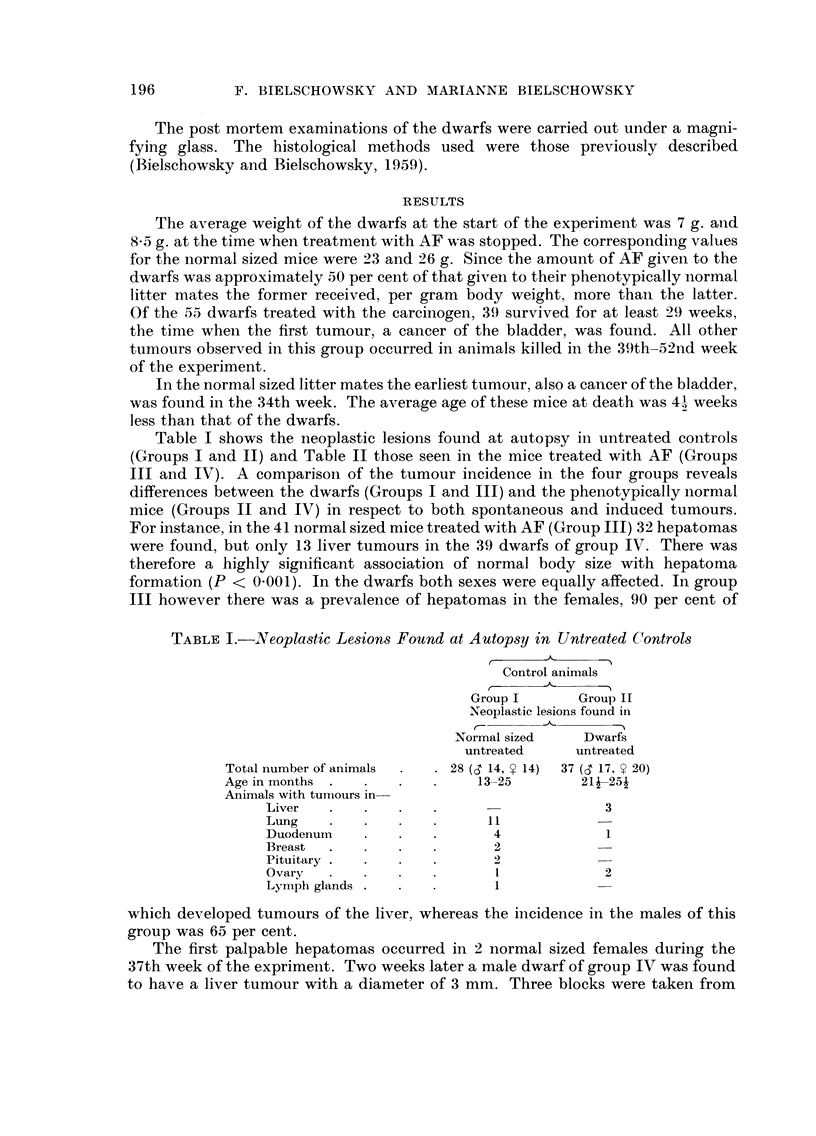

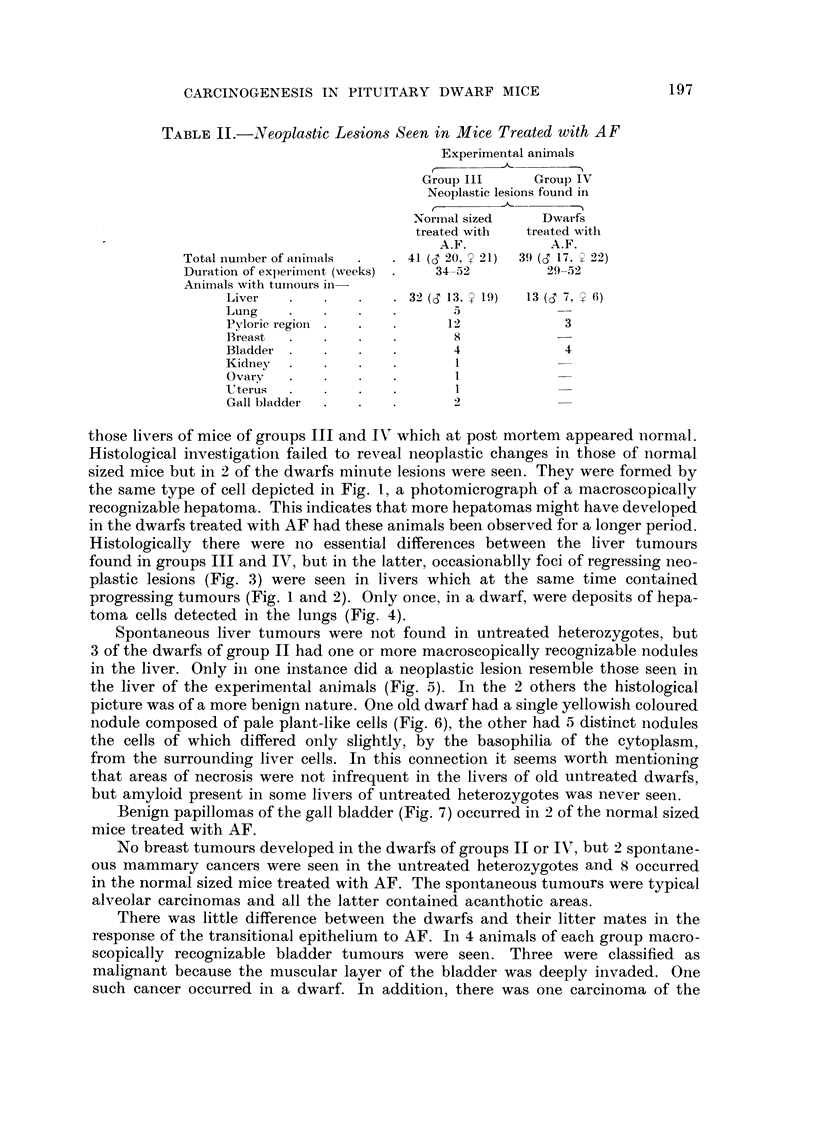

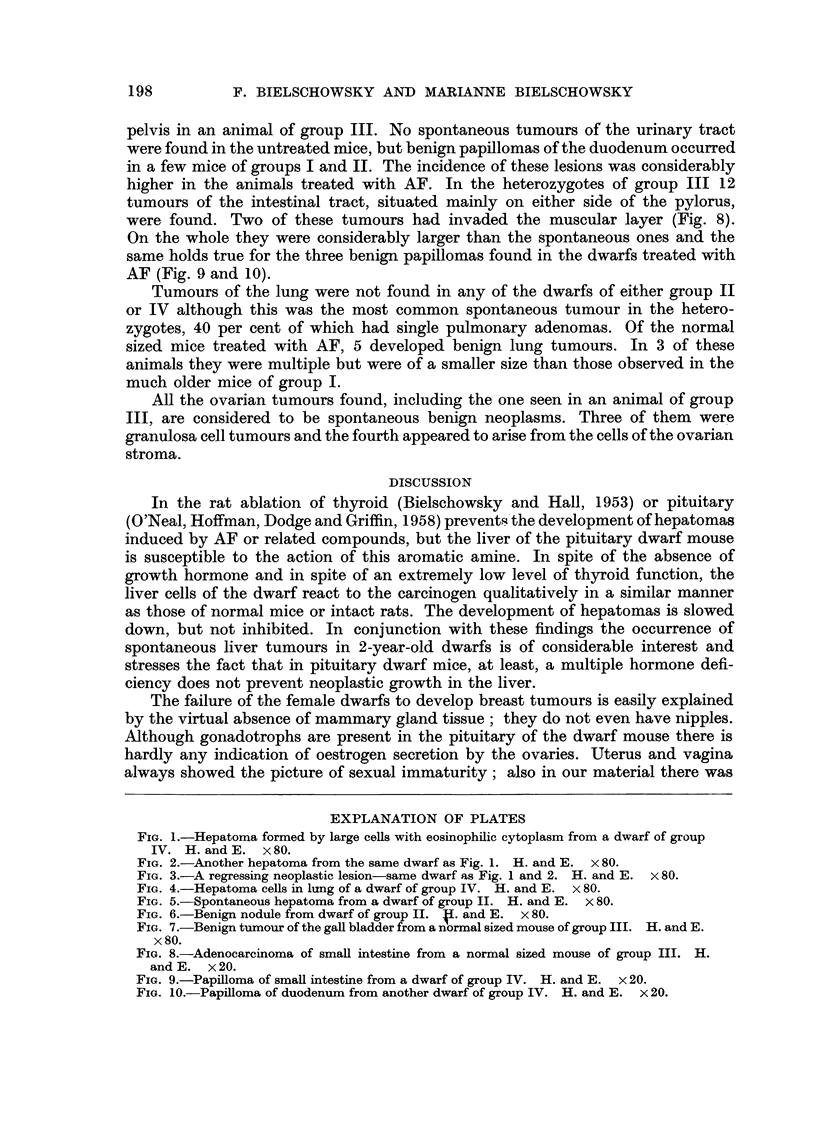

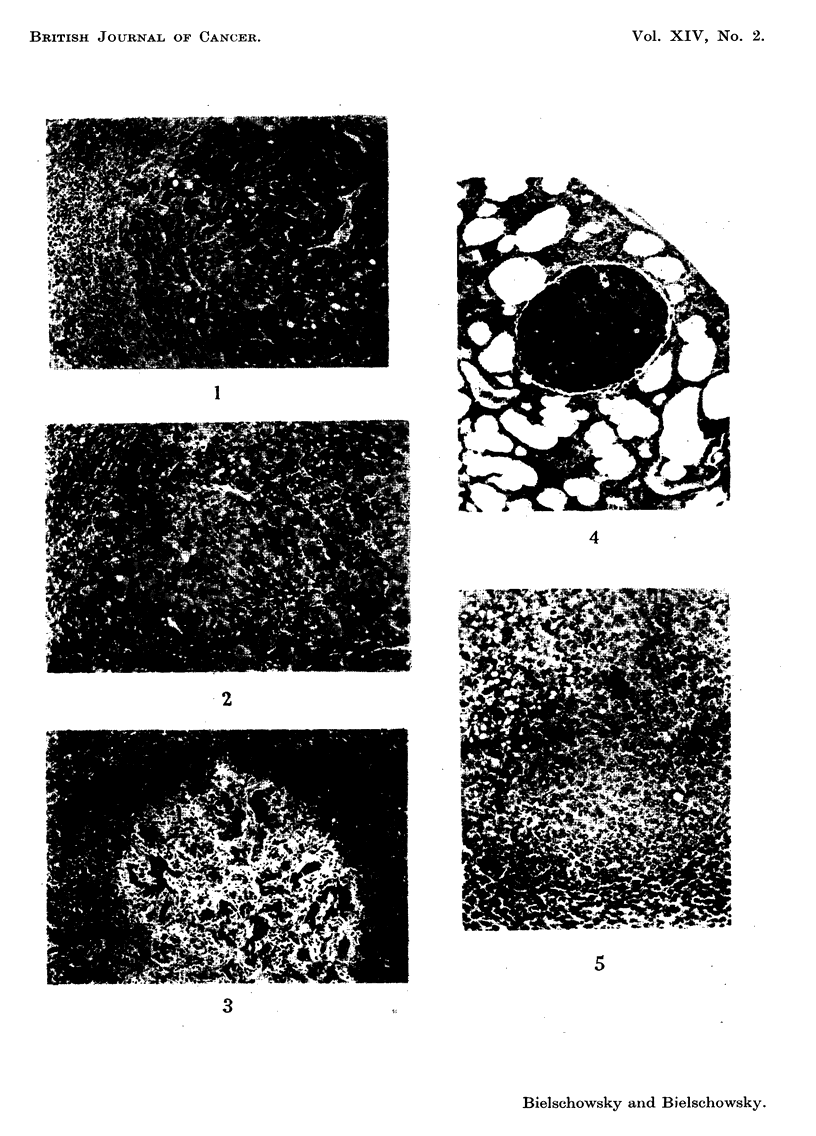

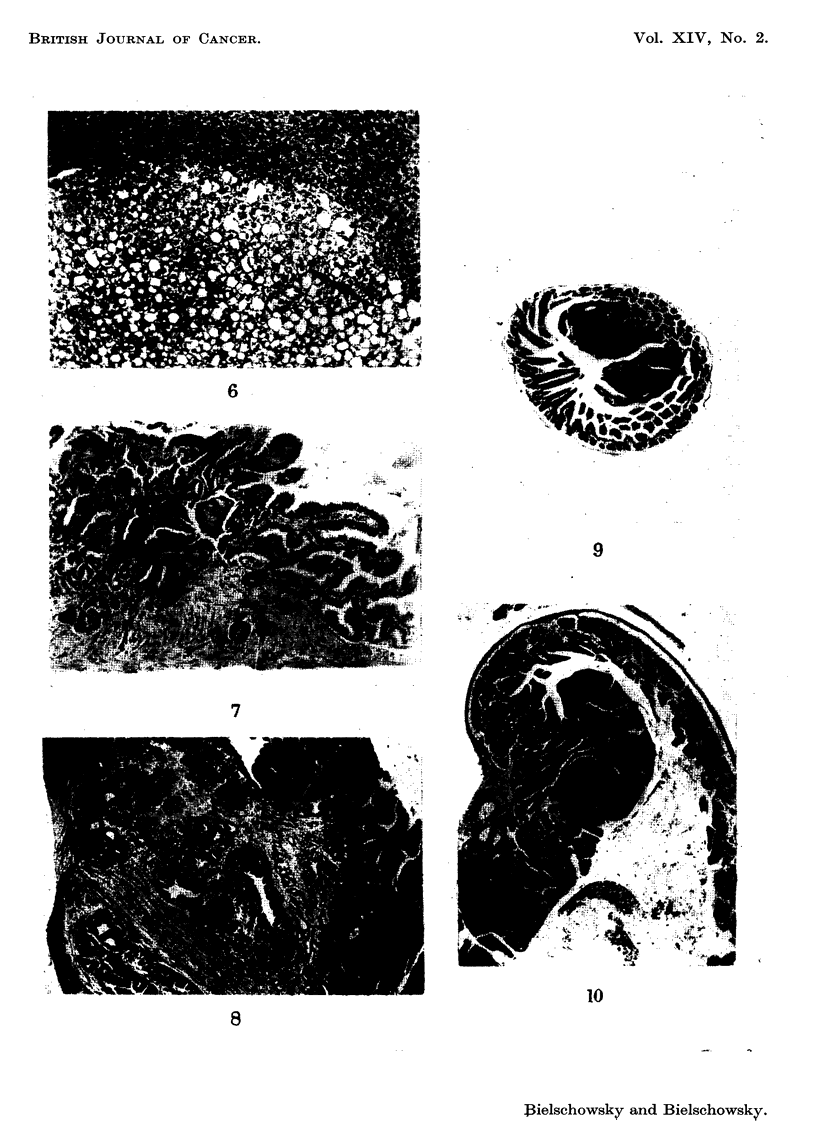

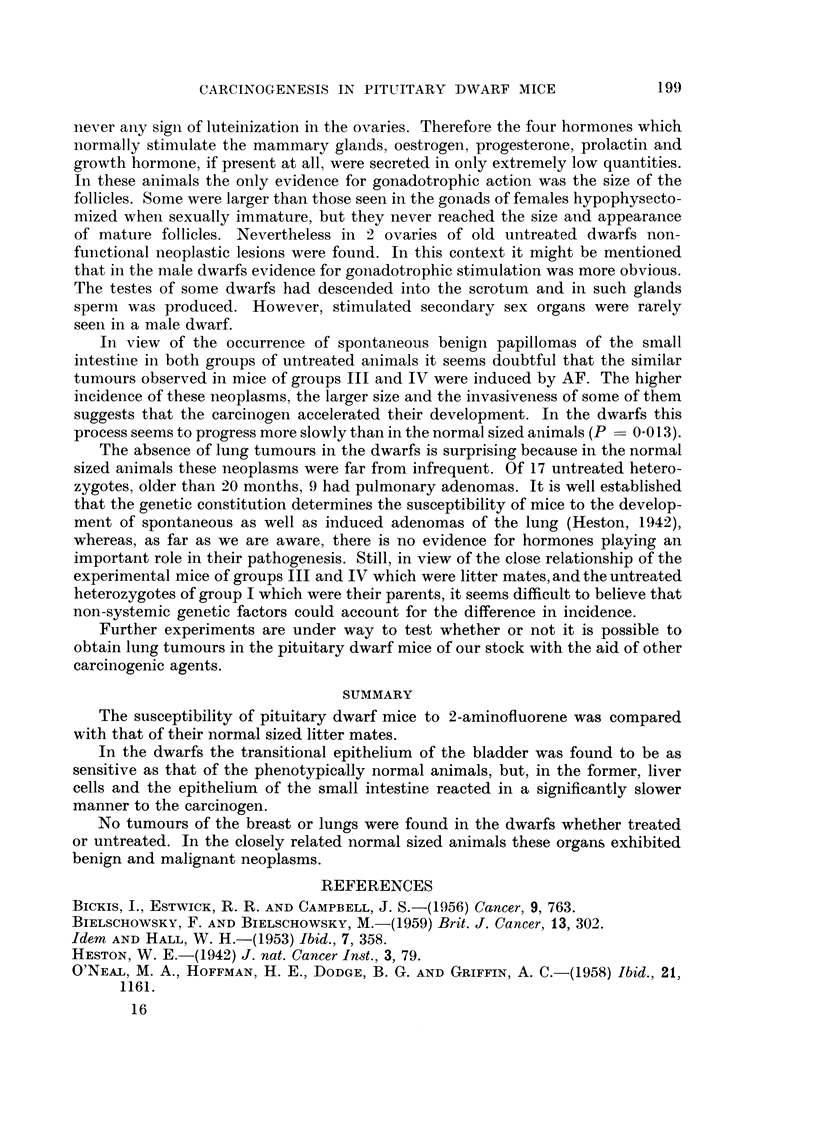


## References

[OCR_00368] BICKIS I., CAMPBELL J. S., ESTWICK R. R. (1956). Observations on initiation of skin carcinoma in pituitary dwarf mice. I.. Cancer.

[OCR_00370] BIELSCHOWSKY F., HALL W. H. (1953). Carcinogenesis in the thyroidectomized rat.. Br J Cancer.

